# Stress-induced changes in the expression of antioxidant system genes for rice (*Oryza sativa* L.) and bread wheat (*Triticum aestivum* L.)

**DOI:** 10.7717/peerj.7791

**Published:** 2019-11-29

**Authors:** Anton Ermakov, Aleksandr Bobrovskikh, Ulyana Zubairova, Dmitrii Konstantinov, Alexey Doroshkov

**Affiliations:** 1 The Federal Research Center Institute of Cytology and Genetics, Siberian Branch of the Russian Academy of Sciences (ICG SB RAS), Novosibirsk, Russian Federation; 2 Novosibirsk State University, Novosibirsk, Russian Federation

**Keywords:** Plant science, Gene expression, Mathematical biology, Antioxidant system, Stress responce, Computational biology, Genomics, Molecular biology, Transcriptome meta-analysis, Bioinformatics

## Abstract

**Background:**

Plant cell metabolism inevitably forms reactive oxygen species (ROS), which can damage cells or lead to their death. The antioxidant system (AOS) evolved to eliminate a high concentration of ROS. For plants, this system consists of the seven classes of antioxidant enzymes and antioxidant compounds. Each enzymatic class contains a various number of genes which may vary from species to species. In such a multi-copy genetic system, the integration of evolutionary characteristics and expression data makes it possible to effectively predict promising breeding targets for the design of highly-yielding cultivars. In the plant cells, ROS production can increase as a result of abiotic stresses. Accordingly, AOS responds to stress by altering the expression of the genes of its components. Expression profiles of AOS enzymes, including their changes under stress, remains incomplete. A comprehensive study of the system behavior in response to stress for different species gives the key to identify the general mechanisms of AOS regulation. In this article, we studied stress-induced changes in the expression of AOS genes in photosynthetic tissues for rice and bread wheat.

**Methods:**

A meta-analysis of genome-wide transcriptome data on stress-induced changes in expression profiles of antioxidant genes using microarray and next generation sequencing (NGS) experiments from the GEO NCBI database for rice and bread wheat was carried out. Experimental study of expression changes in short (6 h) and prolonged (24 h) cold stress responses for selected AOS genes of bread wheat cultivars Saratovskaya29 and Yanetzkis Probat was conducted using qPCR.

**Results:**

The large-scale meta-transcriptome and complementary experimental analysis revealed a summary of fold changes in the AOS gene expression in response to cold and water deficiency for rice and bread wheat.

## Introduction

Typically, plant cell metabolic processes such as aerobic respiration and photosynthesis produce reactive oxygen species (ROS). However, under stress conditions, the production of ROS is multiplied by many times (Hammond-Kosack & Jones, 1996; Orozco-Cardenas & Ryan, 1999; Apel & Hirt, 2004; You & Chan, 2015), causing the so-called oxidative stress, which leads to a decrease in productivity plants or even their death. Antioxidant system (AOS) protects the cell from ROS. This system is multi-copy, and the number of its genes varies from species to species. In particular, about 40 genes in the *Arabidopsis thaliana* genome encode antioxidant enzymes (Mittler et al., 2004) that catalyze seven classes of reactions in the cell.

The ROS degradation cascade consists of two consecutive reactions. The first reaction involves the enzyme superoxide dismutase (SOD), reacting directly with superoxide (O_−_^2^), causing a dismutation reaction and resulting in a peroxide (H_2_O_2_). In the second reaction, the hydrogen peroxide can be split further into the water in three different ways: directly by catalase (CAT), by enzymes of the ascorbate-glutathione cycle (Asc-Gsh) and by glutathione peroxidase (GPX) (Rüffer, Steipe & Zenk, 1995). Asc-Gsh cycle consists of ascorbate peroxidase (APX), antioxidants (ascorbate (AsA), glutathione (GSH)) and enzymes which reduce antioxidants: glutathione reductase (GR), dehydroascorbate reductase (DHAR) and monodehydroascorbate reductase (MDHAR). GSH is a tripeptide and together with GPX can restore connections of S-S type. The oxidized GSH is restored by GR (Couto, Wood & Barber, 2016). GPX enzymes catalyze the reduction of peroxide, lipid peroxides and organic hydroperoxides in presence of glutathione as hydrogen donor (Maiorino et al., 1995). These enzymes in comparison with CAT works slowly but with higher affinity, decompose small amounts of peroxide, which is formed in the cell during normal metabolism (Khompatara et al., 2019).

In plants, two groups of stress factors are conventionally distinguished: (i) abiotic stresses are characterized by a system strategy to increase the activity of the AOS components and control the growing number of radicals, up to depletion of the system capacity; and (ii) biotic stresses caused by so-called oxidative burst are characterized by a decrease of antioxidant activity of enzymes in separate plant cells which were infected. Some reviews (Ahmad et al., 2010; Yoshida et al., 2006; Eltayeb et al., 2007) summarize information on the functioning of AOS confirming that regulation of the system components is important for various stress tolerance.

Several studies showed that transgenic plant lines with additional copies of antioxidant protection genes showed higher tolerance to various types of stress than the original lines. Gill & Tuteja (2010) generalized ideas about the effect of enhancing individual components and indicated that plants with transgenic SOD often have an increased resistance to salinity and water stress, plants with enhanced DHAR activity have better drought and salt tolerance, improved activity of peroxidases (APX and GPX) possess multiple spectra of tolerance enhancement, plants with transgenic CAT improved tolerance to salt and cold stress is observed. The similar effect was demonstrated by experiments with cadmium and heat stress (Zhao, Liu & Zhang, 2009), with salt stress (Lu, Liu & Liu, 2007; Yan et al., 2016), with drought and salinity (Negi et al., 2015; Sun et al., 2018), and with cooling (Xu et al., 2014). Wu et al. (2015) and Zhang et al. (2013) showed that plant lines knockout for individual antioxidant protection genes are less tolerant of stress conditions. Also, two cytosolic APX’s genes, *OsAPX1* and *OsAPX2*, play crucial roles in abiotic stress tolerance in rice (Sato et al., 2011; Zhang et al., 2013). Miao et al. (2006) analyzed mutants and GPX-overexpressors for the *AtGPX3* gene. Mutants showed a high rate of water loss and peroxide production in cells during drought, while overexpressors showed the opposite effect. Interestingly, some results for increasing of expression GR in mutants did not show the increase of stress tolerance (Logan et al., 2003; Mahan et al., 2009). This evidence series speaks in favor of the complex nature of the AOS regulation.

A number of studies on *A. thaliana* showed that the transcriptional regulation of antioxidant enzymes correlated with their activity, for example, Tsang et al. (1991) showed this fact for SOD class. The mRNA concentration for all CAT genes increased in response to phosphorus starvation (Kandlbinder et al., 2004). Also, Abercrombie et al. (2008) illustrated that several genes from SOD class are upregulated in response to arsenic treatment: Cu/Zn SOD (AT2G28190, AT1G08830) and copper chaperone for SOD (AT1G12520), but expression of Fe SOD is downregulated. Thus, the mentioned studies confirm that the transcriptional activity of the AOS enzymes can vary significantly in response to stress.

Most experimental studies devoted to the effects of stresses on the AOS components and the associated regulatory systems included limited sets of replicas, stress factors, and time points. However, the great complexity of the AOS regulation described above requires an integrated approach for understanding the mechanisms of its functioning. Therefore, it is essential to investigate the transcriptional response of the AOS in different conditions by large-scale meta-analysis of experimental data.

This article is devoted to the analysis of the AOS genes regulation for the essential agricultural cereals in response to abiotic stress conditions. In the previous study (Doroshkov & Bobrovskikh, 2018) for cereals we classified the genes encoding the key AOS enzymes (SOD, CAT, APX, GPX) directly detoxificating the ROS and peroxide molecules by expression and evolution related characteristics. A set of enzymes characterized by a high conserved evolution and highest mRNA expression level in the root or shoot tissues was highlighted as the core part of the system as opposed to poorly expressed and more evolutionary variable genes which therefore indicate weak functional load. Also, we described the third group of genes which are characterized by a highly conservative evolution in cereals but do not reveal significant levels of expression in normal environmental conditions. We suppose that stress factors may activate these third group of genes which therefore have an adaptive impact in the plant life cycle. In this study, the hypothesis was tested by an extensive meta-analysis of transcriptome data for *Oryza sativa* L. and analysis of transcriptome data and experimental expression profiling for *Triticum aestivum* L. for some genes. We have studied the water deficit and cold responses and found significant stress-induced transcription variations for the AOS genes.

## Materials and Methods

### Phylogenetic analysis of antioxidant genes

Enzyme families (APX, GPX, CAT, SOD, DHAR, MDHAR, GR) were identified for a *O. sativa*, *Hordeum vulgare* and *Chlamydomonas reinhardtii* (used as an outgroup) using the PLAZA 3.0 database (Proost et al., 2014) and *T. aestivum* from Ensembl Plants release 37 (Kersey et al., 2015) using the reciprocal search for the homologues of the corresponding *A. thaliana* genes in the database with a threshold of *E*-value = 10^−7^ BLAST algorithm (Altschul et al., 1990). An analysis of the molecular evolution of the identified genes was carried out using the SAMEM v0.83 (Gunbin et al., 2012) and MEGA6 software (Tamura et al., 2013). The original sample of homologous sequences was subjected to multiple alignment of sequences by the Mafft 6.717 algorithm (Katoh & Toh, 2008) using the BLOSUM 62 matrix (Henikoff & Henikoff, 1992). After that, an expert assessment of the alignment and the allocation of conservative motives was carried out. Models of amino acid substitutions were calculated based on multiple alignment using the Modelestimator algorithm (Arvestad, 2006). Phylogenetic trees were calculated on the basis of the replacement model using algorithms: FastTree 2.1.1 (Arvestad, 2006)—for evaluating the topology and Phyml (Guindon & Gascuel, 2003; Sandie & Aris-Brosou, 2014)—for final optimization.

### Meta-analysis of genome-wide transcriptome data

#### Sampling and clustering of experiments

The GEO Datasets (Edgar, Domrachev & Lash, 2002; Barrett et al., 2012) were used as a source of data for the expression profiles of antioxidant genes for rice (*O. sativa*) and bread wheat (*T. aestivum*) obtained in control and stress (water deficiency and cold) conditions (Barrett et al., 2005). The initial sample consisted of 22 experiments for rice (GSE21651, GSE23211, GSE24048, GSE25176, GSE26280, GSE37940, GSE38023, GSE41647, GSE4438, GSE54466, GSE64576, GSE65024, GSE65025, GSE67373, GSE6901, GSE71680, GSE74465, GSE78972, GSE80811, GSE81462, GSE83378, GSE83912), and seven experiments for wheat (GSE23889, GSE30436, GSE31762, GSE42214, GSE45563, GSE47090, GSE79522), including data from microarrays and NGS platforms (see Table S1).

Each experiment contains genes expression profile corresponding to control and to one or several variants of stress conditions that differ in time and/or intensity of cold and/or water deficiency.

From these experiments, the expression profiles of antioxidant genes for seven classes of AOS enzymes were extracted. NGS data were normalized by the FPKM method. Microarray and NGS data were subsequently normalized to the expression of housekeeping genes: the UBQ10 gene (OS02G06640) was used for rice and the tubulin 4 alpha chain gene (TRIAE_CS42_5BL_TGACv1_405584_AA1330850) was used for bread wheat. Then, for each of the selected antioxidant genes in each experiment, the average expression was calculated for all replicates in control conditions, which was used to normalize the expression values of this gene in both control and stress conditions. The obtained normalized values reflect the amount of deviation of the expression of each gene from its average expression level under control conditions. Thus, for all selected experiments, we obtained a set of replicas consisting of the normalized values of the expression for each gene under study.

For rice, all experimental points were clustered by the Ward algorithm based on the obtained profiles using the Past program (Hammer, Harper & Ryan, 2001). Then, according to the clustering, we selected experiments that were characterized by an explicit stress response by the following rule: if more than 50% of stress replicates fell into a cluster with control replications, then such an experiment was not taken for further analysis, since it contains an implicit response to stress or noisy data. Thus, the final sample for rice included 13 experiments: GSE21651, GSE25176, GSE26280, GSE37940, GSE38023, GSE41647, GSE54466, GSE64576, GSE65024, GSE65025, GSE6901, GSE78972, GSE81462 (Table S1) containing 200 biological replicates, including 70 control and 130 water deficient and cold replicas. The resulting sample also was clustered by the Ward algorithm.

For bread wheat, we did not preselect experiments because of the small sample size. Therefore, we analyzed changing of expression levels of AOS genes in individual experiments and performed statistical analysis between control and stress replicas to investigate statistically significant response AOS genes.

#### Statistical analysis

Clustering and principal component analysis (PCA) was obtained using the Past program (Hammer, Harper & Ryan, 2001). Statistical data processing on clusters for rice and on types of effects inside individual experiments for wheat was carried out using a *t*-test with the Benjamini–Yekutieli correction. Samples of expression characteristics in stress clusters for rice and replicas for wheat were compared against control replicas and *E*-value were calculated. The genes of up- and down-regulation in stress were marked by these values.

### Experimental study

#### Genetic material and growing conditions

For an experimental study, bread wheat *T. aestivum* L. cv. Saratovskaya 29 (S29) and cv. Yanetzkis Probat (YP) were used. The cv. S29 possesses an increased tolerance to stress conditions and the cv. YP, on the contrary, is less tolerant (Doroshkov, Pshenichnikova & Afonnikov, 2011; Doroshkov et al., 2016). Therefore, this pair of genotypes was chosen to study stress-induced changes in the expression of the AOS genes in plant leaves.

Germinated seeds of plants for both varieties were planted in groups of at least six plants of each variety in plastic containers (PET, size 56 × 39 × 28 cm) with drainage and filled by expanded clay balls. Plants were grown up to the four-leaf stage in a greenhouse (Laboratory of Artificial Plant Growth) at the Institute of Cytology and Genetics SB RAS under a 18/6 h photoperiod (artificial supplementary lighting by Sylvania SHP-TS 600w, illumination of plants was carried out as 15–20 × 10^3^ lx); the temperature condition was maintained at 14–16 °C at night and 20–23 °C during the day. Watering with Knop solution (Hershey, 1994) was carried out daily, until complete saturation of the substrate. Knop solution with additional microelements was used for watering once a week.

#### Stress experiment design and sampling

For stress-modeling experiment for each experimental group we selected containers in which at least four to five plants of each variety grew at the same stage of development. Selected containers with wheat plants were simultaneously either subjected to cold stress (stress groups) or left without stress exposure in described previously growing conditions (control group). Cold stress was simulated by placing containers with plants of stress groups in a climatic chamber with constant temperature +4 °C and same as in greenhouse day/night lighting conditions for six (short-term) and 24 h (long-term cold stress conditions).

Each plant material sample was obtained by pooling of the third leaf middle part from three plants of same cv. grown in one container. The total weight of each sample was 100–200 mg. In this way three samples of plant material per each experimental group of plants was taken. After collection, the samples were immediately frozen in liquid nitrogen and stored at −70 °C until RNA isolation. Sampling was carried out simultaneously for the control and both stress groups of plants.

#### RNA isolation and RT-qPCR analysis

Total RNA was extracted from the collected samples using Plant RNA MiniPrep Kit (Zymo Research, Irvine, CA, USA) with additional treatment with RNase-Free DNase Set (QIAGEN, Hilden, Germany) according to the manufacturers instructions and protocols. Evaluation of the quantity and purity of RNA was performed on a Nanodrop 2000 spectrophotometer (Thermo Scientific, Wilmington, DE, USA), integrity was assessed using agarose gel electrophoresis. For cDNA synthesis, an OT-M-MuLV-RH Reagent Kit (Biolabmix, Novosibirsk, Russia) was used, the reaction was performed according to the manufacturer’s standard protocol with oligo(dT)_16_ primers in 20 μl per reaction volume.

For the experimental analysis, nine orthologs from key classes of APX, SOD and CAT enzymes were selected. The nomenclature of selected genes corresponds to our previous paper (Doroshkov & Bobrovskikh, 2018).

The primers were designed complementary to consensus sequence for all three homeologues of the CAT, APX and SOD genes. Design of the primers was made with the online service PrimerQuest Tool (Integrated DNA Technologies, Coralville, IA, USA) based on the Primer3 algorithm (Untergasser et al., 2012) with default conditions (Ta_opt_ = 60 °C, option “qPCR 2 primers with intercalating dyes”), and we also used the database of nucleotide sequences EnsemblPlants release 37 (Kersey et al., 2015). The amplicon location was within 2,000 bp from the 3′-end and the last intron-exon boundary was chosen in cases where this was possible. Specificity of the primers was checked in silico using the database of nucleotide sequences (NCBI Resource Coordinators, 2019) and algorithms NCBI BLAST, Primer-BLAST (Altschul et al., 1990; Ye et al., 2012). The gene encoding 18S rRNA was used as a reference gene. The primer sequence for the 18S rRNA gene was used from Beales et al. (2007). The specificity test was carried out in a similar way as for the other primers. The sequences of primers used in this work are shown in Table 1.

**Table 1:** The list of clades and correspondent orthological genes for analyzed cereal species (barley, maize, rice and bread wheat) used for further experimental verification. The column “Gene groups” indicates names of clades following the terminology proposed in Doroshkov & Bobrovskikh (2018) based on the data on evolutionary conservation in genes; the column “Sequence” contains information about primers designed for experimental analysis on bread wheat. The table also contains primers of the reference gene 18s rRNA.

Gene group	Target ensembl loci	Sequence (F, forward; R, reverse)	Product size (bp)	Associated barley sequences	Associated maize sequences	Associated rice sequences
APX D	5DL_433012_AA1398100 U_642188_AA2112960 5AS_392962_AA1266880	F-GGACATCACAGTGGCTCAAG R-GATACACCCTTAGGAGGGTCA	231	HV158476G00020	None	OS12G07830 OS12G07820
APX E	4DL_342793_AA1122380 4AS_307333_AA1019630 4BL_320270_AA1033310	F-GGATATTGTTGCCCTCTCTGGT R-GGGTCAGTCAGCAGGGTTT	183	HV40855G00010	ZM01G11990 ZM09G23350	OS03G17690
APX F	U_641465_AA2095890 2BS_146384_AA0464000 2DS_179678_AA0608540	F-GGTCACACCCTGGGAAGAT R-CGGTCGGCAACTGAAGAAG	139	HV56215G00010	ZM02G41460	OS07G49400
CAT A	7AL_556567_AA1765730 7DL_602975_AA1973160 7BL_576925_AA1860110	F-ATGCAGGAGAACTGGAGAATAG R-TGCCTGTAGTTGAGTGGAATG	107	HV7888G00010	ZM05G16210	OS06G51150
CAT B	5AL_376544_AA1238210 4BL_320451_AA1039710 4DL_343177_AA1131150	F-CCACTACGACGGGCTCAT R-CGGGCTGCTTGAAGTTGTT	170	HV71788G00010	ZM01G02380	OS03G03910
CAT C	U_640838_AA2076560 6DS_545065_AA1749890 6BS_513206_AA1634480	F-GAGGATCAAGAAGGAGAACGAC R-GCACAAAGTTTACACAGAACGG	186	HV1561906G00010	ZM04G41390	OS02G02400
SOD A	4AL_289135_AA0965570 7AS_571265_AA1846380 7DS_625535_AA2065460	F-GCCCAATCCCATCTGACAA R-TACTCAGCCCTTCGATTCTCA	151	HV56996G00010	ZM09G06320	OS06G02500
SOD B	7AS_570531_AA1837180 4AL_291448_AA0994630	F-TTTCAGGGAGGAGTTCATGC R-ACAGTATGCCAAGACACAAGG	156	HV2547265G00020	ZM06G08270 ZM09G05010	OS06G05110
SOD E	2AS_114204_AA0365000 2BS_148548_AA0493640 2DS_179233_AA0605480	F-CGCCATGCTGGTGATCTT R-CAGCTCATGTCCACCCTTG	164	HV160480G00010	ZM07G29600 ZM09G21130 ZM01G15520	OS03G22810 OS07G46990
Reference: 18s rRNA		F-ATACGTGCAACAAACCC R-CTACCTCCCCGTGTCA	288			

Oligonucleotides were synthesized in the Biosset Ltd. (Novosibirsk, Russia). Verification of the amplicon specificity was carried out by imaging on agarose gel electrophoresis and building a melting curve using a Lightcycler 96 (Roche Diagnostics, Indianapolis, IN, USA). Optimization of the amplification conditions was not required.

Real-time PCR was performed using a standard three-step protocol on a Lightcycler 96 Instrument (Roche Diagnostics, Indianapolis, IN, USA) with low-profile 96-well plates (#PCR-96-LP-FLT-C; Corning Axygen Scientific, Union City, CA, USA), Ultra-Clear optically transparent films (#UC-500; Corning Axygen Scientific, Union City, CA, USA), and BioMaster HS-qPCR SYBR Blue reagent kit (2×) (Biolabmix, Novosibirsk, Russia). Pre-denaturation was 7 min at 95 °C; denaturation was 18 s at 95 °C; primer annealing was 20 s at 60 °C; elongation was 15 s at 72 °C with detection at the end of the cycle; 45 cycles. The total volume of one qPCR reaction was 20 μl. One qPCR reaction contains two μl of diluted with water (1:5) cDNA and forward/reverse primers to final concentration 0.25 μM each. The reaction with the addition of ddH_2_O instead of cDNA was used as a negative control (NTC). Three biological and three technical replicates were used in each qPCR experiment.

#### Relative expression calculation

The genes of interest (GOI) relative expression was determined by ΔΔ*C_q_* method. qPCR data represent the differential expression of AOS genes under cold stress conditions. For this the raw data from LightCycler 96 Instrument was analysed by LinRegPCR software (Ruijter et al., 2009) to determine *C_q_* and efficiency of reactions and then relative ratio of GOI expression in stress conditions was calculated according proposed by Pfaffl (2004) multiple samples efficiency corrected mathematical model (see Eq. (1)) in LibreOffice Calc (The Document Foundation, Berlin, Germany).

(1) $${\rm{Ratio}} = \,{{{{({E_{{\rm{target}}}})}^{\Delta{C_{q\,{\rm{target}}}}\left( {{\rm{MEA}}{{\rm{N}}_{{\rm{control}}}} - \,{\rm{MEA}}{{\rm{N}}_{{\rm{treat}}}}} \right)}}} \over {{{({E_{{\rm{reference}}}})}^{{{\Delta }}{C_{q\,{\rm{reference}}}}\left( {{\rm{MEA}}{{\rm{N}}_{{\rm{control}}}} - \,{\rm{MEA}}{{\rm{N}}_{{\rm{treat}}}}} \right)}}}}$$

Further data analysis was performed with R (R Development Core Team, 2018), R-studio IDE (RStudio Team, 2015) and xlsx R package (Dragulescu & Arendt, 2018). The plots representing expression ration were generated with RColorBrewer (Neuwirth, 2014), ggplot2 (Wickham, 2016) and ggpubr (Kassambara, 2018) R packages.

## Results and Discussion

### Identification of the orthological groups for the AOS genes

Phylogenetic analysis allowed estimating the composition of orthological groups of antioxidant genes and to comparing of stress-induced changes in AOS genes between rice and bread wheat. Reconstructed phylogenetic trees are presented in File S1.

The analysis of molecular evolution revealed 31 orthological groups (clades) of genes for seven classes of the AOS enzymes (eight for APX, five for GPX, seven for SOD, three for CAT, two for GR and DHAR, four for MDHAR). Reconstructed phylogenetic trees (File S1) allow detecting the difference between the AOS genes in dicots and monocots and showing that the genes from APX (B, C, D), APX (E, F), GPX (D, E) arose as a result of duplication in monocots. At the same time, for the gene AT1G63460 (GPX C), the monocots orthologous genes were not detected, and for the GPX A, B (AT2G25080, AT4G31870, AT2G43350, AT2G31570) orthologs were found only in *O. sativa*. Table 1 summarizes data about clades and correspondent orthological genes the cereal species used for further experimental verification.

### Identification of water deficiency and cold stress-induced changes in AOS genes expression profiles for rice and bread wheat according to a meta-analysis of transcriptome data

#### Cold and water deficiency response experiments for rice

**Figure 1:**
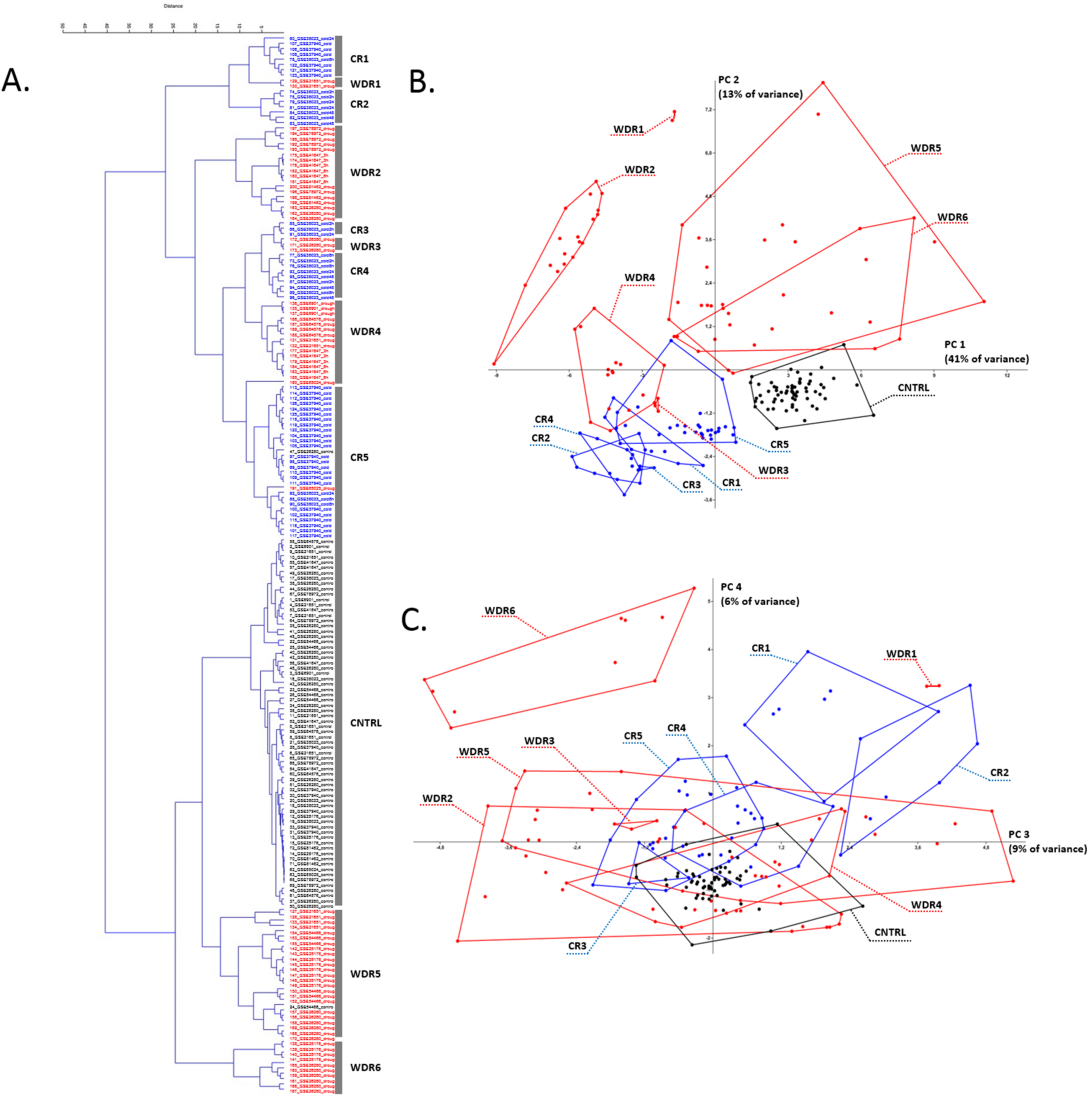
Transcriptome meta-analysis of data on stress-induced changes in the AOS rice genes. The cluster analysis (A) and the principal component analysis (B and C) scatterplots are given. Next, to the component axes, the contribution of the component into all variance is indicated. The clusters are shown with broken stick and are marked with captions in the PCA scatterplots (CR is for cold response, WDR is for water deficiency response). The color of the dots reflects the nature of repetition (black is for control experiment, red is for water deficiency response, blue is for cold response).

For rice, as a result of selection and preprocessing of experiments outputs (see detailed description in “Materials and Methods”) and their further cluster analysis, the replicates were separated into a control cluster, five cold response clusters (CR), and six water deficiency response (WDR) clusters (Fig. 1A; Table S2). The PCA showed that WDR more often combines experiments into clusters, but at the same time, CR clusters are more compact and even all together occupy an area similar in size to the control cluster (Figs. 1B and 1C). The smaller scatter of points inside the clusters and the positions of the clusters relative to each other indicates a more systemic response of the AOS to cold stress. Also, most often, replications from different experiments were combined into one cluster, which shows the similarity of their response to stress and indicates the characteristic behavior pattern of antioxidant genes.

##### Description of CR clusters

**CR1** features a late response to stress and contains two replications of the GSE38023 experiment (shoot, 1× cold 8 h and 1× cold 24 h) and six replications of the GSE37940 experiment (leaves, cold stress 48 h).
**CR2** characterizes an exposure time independent response to stress and contains seven replications of the GSE38023 experiment (leaves, 2× cold 2 h, 2× cold 24 h, 3× cold 48 h).
**CR3** characterizes an exposure time independent response to stress and contains three replications of the GSE38023 experiment (shoot, 2× cold 2 h, 1× cold 24 h).
**CR4** characterizes an exposure time independent response to stress and contains nine replications of a single GSE38023 experiment (shoot, 2× cold 2 h, 3× cold 8 h, 1× cold 24 h, 3× cold 48 h).
**CR5** is the largest cluster represented in most by experiment GSE37940 (shoot, 3× C418 cold stress 2 h, 3× C418 cold stress 6 h, 3× C418 cold stress 12 h, 3× C418 cold stress 24 h, 3× K354 cold stress 2 h, 3× K354 cold stress 6 h, 3× K354 cold stress 12 h, 3× K354 cold stress 24 h), as well as the GSE26280 experiment (1× normal leaves at booting stage, control), the GSE38023 experiment (leaves, 2× cold stress 8 h, 1× cold stress 24 h), and the GSE65025 experiment (1× leaves OSHSFA2e water deficiency).

Thus, among the CR1-CR5 clusters, there is a significant heterogeneity for the GSE38023 experiment, whose replicates occur in each cluster, with repetitions of different duration of exposure to cold stress combined (for example, CR4 cluster), and stresses of the same duration, on the contrary, in different clusters (for example, CR2 and CR4 clusters carry two replicas of 2 h cold). It is noteworthy that the replications of the GSE37940 and GSE38023 experiments in the largest CR5 cluster have different cold exposure times but are clustered together. This clustering may indicate a typical behavior for profiles of antioxidant genes in cold stress of different exposures.

##### Description of WDR clusters

**WDR1** is a small cluster containing two replications of the GSE21651 experiment (water deficiency tolerant leaves 24 h).
**WDR2** is represented by 18 replications from four experiments: GSE78972 (leaves, six replics: 3× Long Day Drought and 3× Short Day Drought), GSE41647 (Dagad deshi seedlings, 3× 3 h drought and 3× 6 h drought), GSE81462 (shoot, three reps drought different genotypes: ZH11D, osbzip23D, OX-OSbZIP23D), GSE26280 (3× drought treated leaves at panicle elongation stage).
**WDR3** is represented by the GSE26280 experiment (3× Drought treated young panicle at booting stage).
**WDR4** is represented by experiment GSE6901 (seedlings, 3× drought), GSE64576 (leaves, 2× drought wild-type, 2× drought overexpression), experiment GSE21651 (2× salt-sensitive leaves 24 h), and experiment GSE41647 (seedlings IR20, 3× 3 h drought, 3× 6 h drought), GSE65024 (leaves, 1× OSbHLH148 drought).
**WDR5** is represented by experiment GSE21651 (leaves, 2× salt-tolerant drought 24 h, 2× sensitive drought 24 h), experiment GSE25176 (flag leaves, 2× IRAT109 drought (D3), 2× ZS97 drought (D1), 2× ZS97 drought (D2), 2× ZS97 drought (D3)), experiment GSE26280 (3× dured treated leaves at tillering stage, 3× drought treated leaves at booting stage), experiment GSE54466 (fresh leaves, 3× WT plants under drought stress condition, 3× osnox2 mutant under drought stress condition, 1× osnox2 mutant under normal growth condition).
**WDR6** is represented by the GSE25176 experiment (flag leaves, 2× IRAT109 (D1), 2× IRAT109 (D2)), and the GSE26280 experiment (roots, 3× Dried treated roots at tillering stage, 3× Drought treated roots at panicle elongation stage).

Water deficiency response clustering in the case of the GSE26280 experiment demonstrates a significant dependence on tissue (WDR2 has 3× drought treated leaves at panicle elongation stage, WDR6 has 3× drought treated roots at panicle elongation stage) and development stage (WDR2 has 3× drought treated leaves at panicle elongation stage, WDR5 has 3× drought treated leaves at tillering stage, 3× drought treated leaves at booting stage). The GSE41647 experiment clustered according to the plant genotype, but not the exposure time: the WDR2 cluster contained the Dagad deshi genotype (3 and 6 h drought), the WDR4 contained the IR20 (3 and 6 h drought). The GSE21651 experiment clustered by resistant and stress-sensitive genotypes. For example, drought-tolerant leaves 24 h occurred in WDR1, drought-sensitive leaves 24 h occurred in WDR5. Such a nature of WDR-clustering may indicate a large regulatory variability of the antioxidant genes expression profiles in the stress response, depending on the genotype, organ, and stage of organ development.

#### The contribution of individual AOS genes in CR and WDR

For rice, we investigated 35 genes encoding AOS components. In CR clusters, we observed 31 AOS genes, which characterized by explicit up- or down-regulation. Also, we obtained eight genes (marked below by asterisk), which are significantly downregulated in all cold-response clusters.

For CR clusters, the following genes are *upregulated*:

**APX:** *OS04G14680*
**GPX:** *OS04G46960*
**GR:** *OS10G28000*
**MDHAR:** *OS08G44340*

For CR clusters, the following genes are *downregulated*:

**APX:** *OS04G35520*, *OS02G34810*, OS12G07820*, *OS12G07830*, OS07G49400*, *OS08G43560*
**CAT (all genes)**: *OS06G51150*, *OS03G03910*, *OS02G02400*
**DHAR (all genes):** OS05G02530*, OS06G12630*
**GPX:** *OS06G08670*, *OS03G24380*, OS11G18170*
**GR:** OS02G56850*, OS03G06740*
**MDHAR:** OS09G39380*, *OS02G47800*, *OS02G47790*, *OS08G05570*
**SOD (all genes):** *OS04G48410*, *OS06G05110*, *OS06G02500*, *OS03G22810*, *OS07G46990*, *OS08G44770*, *OS05G25850*.

In WDR clusters, we observed ten AOS genes, which characterized by clear up- or down-regulation. Also, we obtained three genes, two of them were downregulated (marked below by asterisk), and gene OS06G12630 (DHAR) has a mixed regulation.

For WDR clusters, the following genes are *upregulated*:

**GR:** *OS10G28000* (also observed for CR)
**MDHAR:** *OS08G44340* (for five from six WDR clusters, which is also observed for CR)

For WDR clusters, the following genes are *downregulated*:

**APX:** *OS08G41090*, *OS07G49400* (also observed for CR), *OS08G43560* (also observed for CR), *OS12G07830* (also observed for CR),
**CAT:** *OS03G03910*, OS02G02400* (for both also observed for CR),
**GPX:** *OS06G08670*, OS11G18170* (for both also observed for CR),
**MDHAR:** *OS02G47800*, *OS02G47790*,
**SOD:** *OS03G11960*, *OS03G22810* (for all also observed for CR).

We compared upregulated genes during stress response with their characteristics studied in Doroshkov & Bobrovskikh (2018). Gene OS04G14680; APX H (highly conservative with low expression level) significantly upregulated in four clusters of stress-response (CR1, CR5, WDR1, WDR6). Gene OS06G51150; CAT A (highly conservative, expressed highly in roots) significantly upregulated in three WDR clusters (WDR1, WDR2, WDR5). Gene OS04G46960; GPX E (highly conservative with high expression level) significantly upregulated in CR clusters (CR1, CR2, CR4, CR5). SOD genes OS04G48410; SOD G (low conservative); OS07G46990; SOD E (highly conservative with high expression level); OS05G25850, SOD C (low conservative with low expression level) are significantly upregulated in WDR clusters (WDR1, WDR5, WDR6; WDR1, WDR2, WDR5, WDR6; WDR1, WDR2, WDR5, WDR6, accordingly). Also, some genes that were not characterized in the previous study were upregulated: OS10G28000; GR A (eight clusters); OS05G02530, DHAR B (WDR5, WDR6); OS08G44340, MDHAR A (seven clusters). So, four of six genes which founded upregulated are highly conservative, which can indicate the importance of this characteristic for pre-selection of important AOS genes

#### Cold and water deficiency response experiments for bread wheat

For bread wheat (*T. aestivum*) due to the small sample size (seven experiments), we did not preselect experiments. Therefore, we analyzed separated experiments with specific stress conditions separately (Table S3).

By analyzing the microarray experiments, we obtained data only for some AOS genes because platform GPL3802 had only some samples of AOS genes. Also, microarray probe measure total expression of all homeological genes, which imposes additional restrictions on measurement accuracy. Platform included probes for APX B, APX G, GPX D, SOD C, SOD F, CAT A, CAT B, GR A, GR B, DHAR B. For the other hand, analysis of NGS experiment GSE79522 provide data about each copy of AOS genes in bread wheat.

Stable and most interesting data about regulation of AOS genes during stress was obtained from experiments GSE79522 and GSE23889. In water-deficient experiments GSE79522 we revealed a significant upregulation of CAT A, APX G, SOD D, CAT C, MDHAR A significantly and downregulation of APX F and DHAR A. Analysis of cold stress treatment of experiment GSE23889 showed significantly downregulation of following genes: CAT A, CAT B, GR A, GR B, DHAR B between control and stress replicas. By analyzing the expression of antioxidant genes depending on the time of acclimatization on GSE23389 (see link) (2, 14, 21, 35, 42, 56, 70 days) was significantly revealed upregulation for GPX-E (in most cases for spring Manitou), SOD-F (some time points for every genotype), GR-B for winter Manitou (2 days, 21 days) and spring Manitou (2 days), DHAR-B (in most cases of winter Manitou and spring Manitou and downregulation of GR A for spring Norstar (2, 35, 56, 70 days).

So, the following analysis on bread wheat data gives an incomplete picture about regulation of AOS genes in stress conditions. Therefore, we decided to study AOS genes in our experiment, which include different groups of genes of AOS of bread wheat.

### Experimental study of cold-induced changes in AOS genes expression for bread wheat

The experimental study of cold-induced changes in AOS genes expression for bread wheat included nine orthologs from key enzymatic classes CAT, APX, and SOD. Inside each class representatives of orthological groups, the choice was partly based on the level of expression in normal conditions and the index of evolutionary conservativity for the respective genes. Orthological groups of genes were labeled according to the phylogenetic analysis presented above (see Table 1). Also, the Table 1 contains the Ensembl ID of the genes, ortholog group primers sequences, and correspondent orthological genes for rice and arabidopsis. For SOD and APX classes, the experimental analysis included two highly conservative (APX E, APX F, SOD B, SOD E) representatives and a low conservative one (APX D and SOD A). For CAT class, representatives of all three clades (CAT A, CAT B, CAT C) were highly conserved.

**Figure 2:**
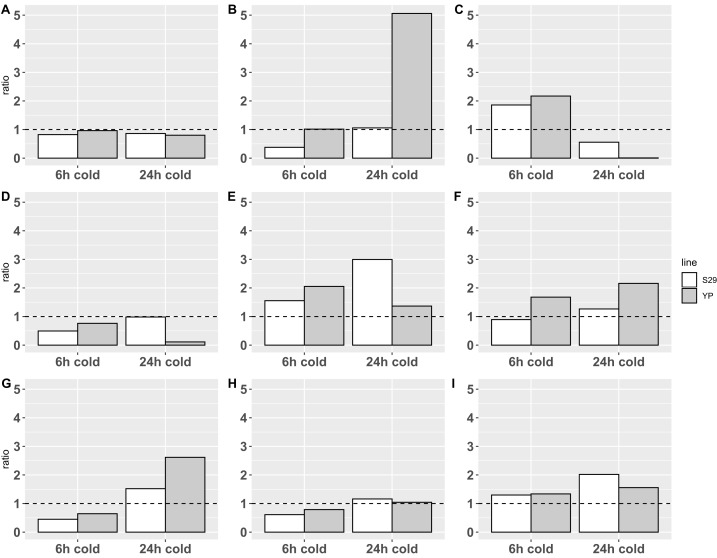
Relative expression data obtained by real-time PCR. The expression change ratio was calculated by ΔΔCq model with efficiency correction (Pfaffl, 2001, 2004; Ruijter et al., 2009) and depicted with barplots. Expression of genes in control group are depicted by plot line *y* = 1. (A–C) Catalases CAT A, B, C; (D–F) APX D, E and F; (G–I) SOD A, B and E.

Plant experimental groups of cultivars S29 and YP were either subjected to cold stress during 6 or 24 h (stress groups) or grown in normal conditions (control group). Consequently, the total RNA isolated in biological triplicates was used to analyze the differential expression of genes by RT-qPCR method (see Fig. 2; Table S4).

Statistical analysis on ΔC_*q*_ data with 2-way ANOVA revealed an impact of cold treatments on expression of all genes except CAT A: *p*-value < 0.0001 for CAT B and C, *p*-value < 0.05 for APX E and SOD’s, *p*-value < 0.1 for APX D and F. Using different cultivars has an impact on the expression of CAT B (*p*-value < 0.05) only. Revealed with this analysis interaction of different cold treatment times and varieties factors affects the expression of CAT B, C and APX D, E genes (*p*-value < 0.05), but not CAT A, APX F and SOD’s.

The analysis of calculated expression ratio for SOD genes group, showed that SOD A (Fig. 2G), which is a low-conservative representative, retains the overall pattern of reaction in both cultivars downregulating at 6-h treatment (0.45/0.65 for S29/YP) and upregulating during 24-h cold treatment (1.52/2.62 for S29/YP). SOD B (Fig. 2H) in both cultivars is downregulated (0.61/0.79 for C29/YP) during short treatment, and at 24-h cold treatment expression level is restored to norma for YP (1.04) and slightly upregulated for S29 (1.16). SOD E (Fig. 2I) in both cultivars at 6-h cold treatment was upregulated about 0.3 fold (1.30/1.34 for S29/YP) and with an increase in treatment time have a noticeable increasing expression ratio to 2.02 for S29 and a slight (1.55) for YP. In this case, it is possible to note a more intensive reaction for YP (less inhibition during short treatment and stronger activation during prolonged treatment) in contrast to the high-coservative representatives which demonstrate the similarity of the reaction on a short-term treatment and stronger reaction for S29 variety.

For CAT group (all high-coservative) we demonstrated that CAT A (Fig. 2A) slightly downregulates in response to treatment, however, it should be noted that the decrease in the level to 0.8–0.85, which occurs for S29 already in response on a 6 h treatment, for YP happens only with an increase in treatment time to 24 h, still near control group level in 6 h treatment experiment. CAT B (Fig. 2B) with a short-term treatment for S29 strongly downregulates to 0.38 level, and recover expression level (1.06) with an increase in treatment time, whereas the YP does not have changes in the expression ratio on 6 h cold, but in 24 h experiment shows a dramatic increase to 5.06 ratio. CAT C (Fig. 2C) demonstrate similar behavior pattern showing an upregulation for 6 h treatment for both species (1.86/2.17 for S29/YP) and downregulated by 24 h treatment with strong severity for YP again (0.56/0.0064 for S29/YP). For studied by qPCR catalases can be noted that similar reaction patterns observed for both varieties, having a markedly more extent reaction for the less cold tolerant variety YP. Given the high conservativity of all CATs and their important role in the rapid response to oxidative stress, it can be concluded that CATs in this case only react to other changes in cell metabolism (possibly located in different compartments), that is, not act as the primary mechanism of protection against cold.

Analysis of APX group reveals that low-conservative APX D (Fig. 2D) at short-term treatment downregulates in both varieties (0.50/0.76 for S29/YP), whereas at 24 h cold S29 restores the expression (0.99), while YP show a strong down-regulation (0.11). Highly conserved APX E (Fig. 2E) at 6 h cold treatment demonstrates upregulation for both varieties (1.55/2.05 for S29/YP), but with 24 h treatment, the S29 ratio increases to 3.00, while the YP upregulation ratio slightly decreases (1.37). Last of genes, APX F (highly conserved, Fig. 2F) at 6 h cold for S29 downregulates to 0.89 ratio, whereas for YP ratio is increased to 1.68. With an increase in treatment time an upregulation for S29 (1.27) occurs, as in the case of YP (2.16). In general, it can be noted that in response to treatment time, the reaction of highly conserved enzymes in both varieties is similar, whereas a low conservative representative in response to 24 h treatment in the more resistant S29 variety restores the level to 0.98, whereas YP variety shows a strong down-regulation. For a short-term treatment, the highly conserved APX F enzyme can be noted, which is less responsive to treatment in S29. As in the case of catalases, this is related to the low cold resistance of the YP variety.

Summing up, we can observe similar patterns of differential expression and a general tendency to upregulation with an increasing of treatment time for all three groups of enzymes, especially represented in the YP variety for CAT and APX, which is obviously related to the lower cold resistance. However, it is worth paying attention to a more extent reaction for highly conserved SOD (Figs. 2G and 2H) representatives in the more tolerant to cold S29 variety, whereas the low conservative SOD A follows common to the other studied enzymes pattern of behavior. It can be assumed that the analyzed highly conserved enzymes SOD B and SOD E are involved in the primary processes of stabilizing cell metabolism under stress, causing increased resistance to cold, thus deserving more careful study.

It should be noted that SOD E for rice differs in response to both cold and water deficiency. Thus, the expression of the corresponding gene (OS07G469900) in response to drought on average changes by more than two times, which is the maximum change in expression in this meta-analysis.

Baek & Skinner (2003) also showed that wheat acclimatization to cold proceeds with upregulation of the total activity of antioxidant enzymes in varying degrees. During acclimatization, the expression levels of CAT, Cu-Zn SOD, Fe SOD, GPX are relatively stable, but MDHAR, DHAR, GR, and t-APX are highly upregulated. Jiang & Zhang (2002) conducted measurements of the total activity of APX, CAT, SOD, GR in the leaves in response to water deficiency, and during the exposure, the upregulation of the total activity of enzymes was also noted. The review (Foyer & Shigeoka, 2011) shows a number of examples of the fact that over-expression of antioxidant genes in chloroplasts (APX, GPX) increases plant tolerance to photooxidative stress and a number of other abiotic stresses, which stabilizes photosynthetic processes under stress conditions. The concept of the complexity of the stress response in plant metabolism is perfectly illustrated by the work (Ramachandra Reddy, Chaitanya & Vivekanandan, 2004), which notes the complexity of the response to drought and the upregulation of antioxidant genes. Bhatnagar-Mathur, Vadez & Sharma (2008) discusses that a strategy to create transgenic plants with overexpression of antioxidant genes along with plant transgenation on other features (osmolyte synthesis genes, proline and others) is a promising approach to increasing tolerance to abiotic stresses. Gibon et al. (2006) showed that changes in the metabolome and transcriptome do not always correlate with each other and reflect the response at different levels. Thus, on the one hand, consideration of system components in a biochemical context helps to identify general trends in its regulation in response to stress, however, consideration of the characteristics of individual genes is an important task for identifying key enzymatic genes that change significantly during a stress response.

### How the qPCR results match the results of the meta-analysis

The large-scale meta-transcriptome and complementary experimental analysis revealed a summary of fold changes in gene expression in response to cold and water deficiency for rice and bread wheat (see Table 2). For rice, data are provided by clusters of experiments; for bread wheat, data from individual experiments are shown. Comparison data between two species give evidence to a similarity in down-regulation for some orthologs in the stress response, but the difference between orthological groups in upregulation genes in response to stress. Obtained results for rice experiments specifically reflected this trends in the stress response of the system: (i) most AOS genes are downregulated, (ii) the response to cold stress is more homogeneous and consistent than the response on water deficit stress. Due to the microchip specificity and the designation of genes for experimental analysis for the bread wheat, the results mostly complement each other because experimental and bioinformatic analysis contained different AOS genes (intersect is only in CAT A and CAT B).

**Table 2:** Summary of fold changes in gene expression in response to cold and water deficiency for rice and bread wheat revealed by meta-transcriptome and experimental analysis. For rice, data are provided by clusters of experiments; for bread wheat, data from individual experiments are shown. The color of the cell indicates the degree of down- (green) and up- (red) regulation for the corresponding genes; white cells show values close to one, indicating minimal fold change; yellow cells mean no data; gray cells mean no genes.

Enzyme	Clade	*Oryza sativa*											*Triticum aestivum*											
		CR clusters					WDR clusters						CR experiments					WDR experiments						Gene
													S29		YP		23889	30436	31762	42214	45563	47090	79522	
		1	2	3	4	5	1	2	3	4	5	6	6 h	24 h	6 h	24 h								
SOD	A	OS06G02500											0.45	1.52	0.64	2.62							1.25	TRIAE_CS42_4AL_TGACv1_289135_AA0965570
		**0.68**	**0.17**	**0.20**	**0.20**	0.76	**0.12**	**0.20**	**0.51**	**0.24**	1.10	**1.29**											1.28	TRIAE_CS42_7AS_TGACv1_571265_AA1846380
																							1.28	TRIAE_CS42_7DS_TGACv1_625535_AA2065460
	B	OS06G05110											**0.61**	1.16	**0.79**	1.04							0.85	TRIAE_CS42_7AS_TGACv1_570531_AA1837180
		0.80	**0.60**	0.98	**0.77**	0.90	0.81	**0.39**	0.96	**0.37**	1.09	**1.29**											0.87	TRIAE_CS42_4AL_TGACv1_291448_AA0994630
																							1.03	TRIAE_CS42_7DS_TGACv1_622963_AA2047880
	C	OS05G25850															0.99	1.04	0.67	1.06	1.15	1.60	1.02	TRIAE_CS42_2DL_TGACv1_158719_AA0524930
		**0.66**	**0.41**	**0.45**	**0.45**	0.74	**2.85**	**1.68**	0.83	**0.58**	**1.52**	**1.58**											1.06	TRIAE_CS42_2BL_TGACv1_131439_AA0427700
																							0.99	TRIAE_CS42_2AL_TGACv1_94166_AA0293620
	D	OS03G11960																					1.15	TRIAE_CS42_4BL_TGACv1_321588_AA1062590
		0.86	0.92	0.89	0.81	0.88	0.60	**0.25**	1.09	**0.48**	1.05	**0.82**											1.10	TRIAE_CS42_4AS_TGACv1_306564_AA1010270
																							1.03	TRIAE_CS42_4DL_TGACv1_342699_AA1119920
	E	OS03G22810											1.30	2.02	1.34	1.56							1.03	TRIAE_CS42_2AS_TGACv1_114204_AA0365000
		**0.71**	**0.45**	**0.46**	**0.46**	0.74	0.96	**0.54**	**0.40**	**0.58**	0.91	0.89											1.03	TRIAE_CS42_2BS_TGACv1_148548_AA0493640
																							1.15	TRIAE_CS42_2DS_TGACv1_179233_AA0605480
		OS07G46990																						
		0.84	**0.72**	0.66	**0.67**	0.87	**2.37**	**1.75**	0.77	**0.78**	**2.30**	**2.69**												
																						
	F	OS08G44770															0.70	0.92	0.18	1.02	1.05	**0.45**	1.07	TRIAE_CS42_7AL_TGACv1_557214_AA1778230
		**0.56**	**0.28**	**0.48**	**0.42**	0.75	0.61	**0.26**	0.75	**0.54**	1.14	**2.02**											**1.11**	TRIAE_CS42_7DL_TGACv1_602770_AA1968180
																							**1.07**	TRIAE_CS42_7BL_TGACv1_578150_AA1890160
	G	OS04G48410																						
		**0.63**	**0.38**	**0.47**	**0.44**	0.70	**1.51**	**0.65**	1.01	**0.58**	**1.28**	**1.47**												
																						
CAT	A	OS06G51150											0.82	0.86	0.96	0.80	**0.74**	**2.42**	**4.57**	**1.05**	**1.18**	**1.32**	**1.07**	TRIAE_CS42_7AL_TGACv1_556567_AA1765730
		0.74	0.90	0.73	0.80	**0.65**	**1.99**	**1.72**	0.93	0.89	**1.21**	**0.71**											0.84	TRIAE_CS42_7DL_TGACv1_602975_AA1973160
																							**0.88**	TRIAE_CS42_7BL_TGACv1_576925_AA1860110
	B	OS03G03910											0.38	1.06	1.01	5.06	**0.86**	**0.74**	**0.61**	**0.94**	**1.01**	**1.12**	0.78	TRIAE_CS42_5AL_TGACv1_376544_AA1238210
		0.84	0.90	0.78	0.74	**0.83**	0.72	**0.15**	0.92	**0.43**	**0.72**	0.96											0.78	TRIAE_CS42_4BL_TGACv1_320451_AA1039710
																							**1.57**	TRIAE_CS42_4DL_TGACv1_343177_AA1131150
	C	OS02G02400											1.86	0.56	**2.17**	**0.01**							**1.02**	TRIAE_CS42_U_TGACv1_640838_AA2076560
		**0.25**	**0.23**	0.73	**0.54**	**0.51**	**0.21**	**0.26**	**0.27**	**0.35**	**0.62**	**0.80**											1.17	TRIAE_CS42_6DS_TGACv1_545065_AA1749890
																							**1.04**	TRIAE_CS42_6BS_TGACv1_513206_AA1634480
APX	A	OS08G41090																						
		**0.44**	0.92	1.24	0.94	**0.40**	**0.13**	**0.53**	0.66	**0.21**	0.85	**0.79**												
																						
	B	OS02G34810															0.85	0.55	0.23	1.00	0.96	1.61	0.86	TRIAE_CS42_6AL_TGACv1_470886_AA1497540
		0.75	**0.56**	0.77	**0.65**	**0.80**	0.75	**0.15**	0.92	**0.47**	**0.63**	**2.36**											0.88	TRIAE_CS42_6BL_TGACv1_499717_AA1589930
																							1.06	TRIAE_CS42_6DL_TGACv1_526775_AA1691860
	C	OS04G35520																					0.88	TRIAE_CS42_2AL_TGACv1_95116_AA0307060
		**0.53**	0.84	**0.54**	**0.69**	**0.65**	0.52	**0.35**	**0.37**	**0.58**	1.10	**0.48**											0.60	TRIAE_CS42_2DL_TGACv1_158409_AA0517920
																							1.01	TRIAE_CS42_2BL_TGACv1_130399_AA0410390
	D	OS12G07820											0.50	0.98	0.76	**0.11**							1.05	TRIAE_CS42_5DL_TGACv1_433012_AA1398100
		**0.53**	**0.46**	**0.41**	**0.43**	**0.64**	0.93	**0.37**	**0.39**	**0.40**	1.02	1.04											1.04	TRIAE_CS42_U_TGACv1_642188_AA2112960
																							1.23	TRIAE_CS42_5AS_TGACv1_392962_AA1266880
		OS12G07830																						
		**0.57**	**0.31**	0.70	**0.51**	**0.63**	**0.26**	**0.46**	**0.46**	**0.29**	1.01	**0.77**												
																						
	E	OS03G17690											1.55	**3.00**	2.05	1.37							1.21	TRIAE_CS42_4DL_TGACv1_342793_AA1122380
		1.15	1.08	0.92	**0.90**	0.92	0.88	0.90	0.87	**0.69**	1.29	0.95											1.07	TRIAE_CS42_4AS_TGACv1_307333_AA1019630
																							0.60	TRIAE_CS42_4BL_TGACv1_320270_AA1033310
	F	OS07G49400											0.89	1.26	1.68	**2.16**							**0.98**	TRIAE_CS42_U_TGACv1_641465_AA2095890
		**0.44**	**0.51**	**0.31**	**0.32**	**0.52**	0.82	**0.32**	**0.49**	**0.57**	**0.78**	**0.70**											**0.98**	TRIAE_CS42_2BS_TGACv1_146384_AA0464000
																							**0.76**	TRIAE_CS42_2DS_TGACv1_179678_AA0608540
	G	OS08G43560															1.01	1.16	1.20	1.05	1.13	1.15	**0.70**	TRIAE_CS42_7AS_TGACv1_569494_AA1817480
		**0.76**	0.88	0.69	**0.69**	0.83	0.55	**0.14**	**0.50**	**0.53**	**0.65**	**0.70**											**0.66**	TRIAE_CS42_7BS_TGACv1_593978_AA1955430
																							**0.70**	TRIAE_CS42_7DS_TGACv1_624295_AA2060370
	H	OS04G14680																					1.23	TRIAE_CS42_2AS_TGACv1_112531_AA0340050
		**3.41**	1.06	1.25	1.16	**1.72**	**5.16**	**1.55**	0.67	5.00	1.19	**3.81**											1.22	TRIAE_CS42_2BS_TGACv1_146663_AA0470350
																							1.20	TRIAE_CS42_2DS_TGACv1_180302_AA0610730
GPX	A	OS06G08670																						
		0.75	0.82	0.74	**0.71**	**0.83**	**0.26**	**0.16**	0.61	**0.47**	**0.61**	0.89												
																						
	B	OS11G18170																						
		**0.35**	**0.38**	**0.45**	**0.40**	**0.64**	**0.28**	**0.19**	**0.40**	**0.34**	**0.71**	**0.75**												
																						
	C																						0.45	TRIAE_CS42_U_TGACv1_641028_AA2082690
																							1.06	TRIAE_CS42_6DL_TGACv1_526629_AA1688510
																						
	D	OS02G44500															0.87	0.92	2.22	0.90	0.89	1.23	1.15	TRIAE_CS42_2AL_TGACv1_94743_AA0302410
		**1.45**	0.88	**0.57**	0.80	**0.72**	**1.79**	**0.56**	0.61	**1.41**	**0.85**	0.99											1.14	TRIAE_CS42_2BL_TGACv1_130949_AA0420440
																							1.14	TRIAE_CS42_2DL_TGACv1_157956_AA0503330
	E	OS04G46960																					0.92	TRIAE_CS42_4BS_TGACv1_328368_AA1086960
		**1.58**	**1.70**	1.03	**1.25**	**1.08**	1.02	0.85	0.73	0.91	**0.86**	**0.65**											0.92	TRIAE_CS42_U_TGACv1_641855_AA2105830
																							0.92	TRIAE_CS42_4AS_TGACv1_306489_AA1008990
	F	OS03G24380																						
		**0.54**	**0.65**	0.63	**0.60**	**0.79**	**2.17**	1.07	0.63	0.88	**1.28**	**0.90**												
																						
GR	A	OS10G28000															**0.82**	0.59	0.76	0.96	0.72	1.06	0.96	TRIAE_CS42_4DL_TGACv1_344390_AA1146810
		**2.38**	**5.22**	1.07	**2.31**	**1.25**	**4.64**	**1.72**	1.28	1.06	**1.65**	**1.52**											1.07	TRIAE_CS42_4AS_TGACv1_307100_AA1016930
																							2.44	TRIAE_CS42_4BL_TGACv1_321849_AA1066190
	A	OS03G06740																						
		0.69	0.30	0.44	0.38	0.81	0.56	0.17	0.59	0.32	0.99	1.20												
																						
	B	OS02G56850															**0.90**	1.13	2.24	1.04	1.07	1.02	1.59	TRIAE_CS42_6DL_TGACv1_526737_AA1691050
		**0.40**	**0.59**	**0.38**	**0.41**	**0.71**	**1.95**	**1.38**	0.68	0.91	1.10	**0.89**											2.48	TRIAE_CS42_6BL_TGACv1_500983_AA1612210
																							0.82	TRIAE_CS42_6AL_TGACv1_471081_AA1502320
MDHAR	A	OS08G44340																					0.92	TRIAE_CS42_4BS_TGACv1_329408_AA1100960
		**0.39**	**0.34**	**0.28**	**0.25**	**0.59**	0.88	**0.36**	0.35	**0.47**	**0.82**	**1.18**											0.89	TRIAE_CS42_4AL_TGACv1_289606_AA0973980
																							0.99	TRIAE_CS42_4DS_TGACv1_360999_AA1158060
	A	OS09G39380																						
		**2.92**	**3.15**	0.90	**1.72**	**1.22**	**3.89**	1.18	1.01	**1.66**	**1.49**	**0.64**												
																						
	B	OS08G05570																					0.96	TRIAE_CS42_U_TGACv1_642723_AA2122250
		**0.59**	**0.43**	**0.41**	**0.41**	0.67	0.80	**0.34**	**0.45**	**0.50**	1.09	**0.80**											0.61	TRIAE_CS42_7AL_TGACv1_556324_AA1760960
																						
	C	OS02G47800																					1.10	TRIAE_CS42_6BL_TGACv1_499432_AA1582460
		**0.53**	**0.62**	0.81	**0.62**	0.73	**0.44**	**0.22**	0.71	**0.38**	**0.63**	**0.75**											1.21	TRIAE_CS42_6AL_TGACv1_471762_AA1513920
																						
	D	OS02G47790																					1.13	TRIAE_CS42_6BL_TGACv1_499432_AA1582470
		**0.75**	0.74	0.95	**0.74**	0.95	**0.20**	**0.27**	**0.52**	0.93	**0.74**	**0.80**											0.83	TRIAE_CS42_6AL_TGACv1_471762_AA1513930
																					
DHAR	A	OS06G12630																					**0.96**	TRIAE_CS42_7AS_TGACv1_569590_AA1819870
		**0.47**	**0.26**	**0.24**	**0.22**	**0.61**	**0.30**	**0.08**	**0.53**	**0.34**	**0.65**	**1.25**											0.92	TRIAE_CS42_7DS_TGACv1_621452_AA2016070
																							**0.98**	TRIAE_CS42_7BS_TGACv1_591997_AA1927740
	B	OS05G02530																					1.01	TRIAE_CS42_1BS_TGACv1_51062_AA0177560
		**0.45**	**0.22**	**0.32**	**0.23**	**0.64**	0.85	**0.60**	0.75	**0.70**	**1.14**	**1.41**					**0.84**	0.68	0.73	1.10	1.10	1.10	0.56	TRIAE_CS42_1AS_TGACv1_19083_AA0060250
																							0.36	TRIAE_CS42_1DS_TGACv1_80640_AA0251420

**Note:**

Bold entries mean significant changes (*p*-value < 0.05) in the expression (for the method of comparison with control, see the Methods section).

Currently the transcriptome analysis of wheat in response to the cold is mainly related to the study of the features of cold acclimatization processes for spring and winter wheat. Baek & Skinner (2003) studied the reaction of seven antioxidant enzymes to prolonged cold acclimation (1, 2, 4 weeks) and concluded that most enzymes have significantly increased their expression. Characteristics of the expression of enzymes for different classes are similar for winter wheat line NILs 442 and spring wheat line NIL 443, except for CAT, the expression of which is twice higher in control conditions. The study of Laudencia-Chingcuanco et al. (2011) (experiment GSE23889) focuses on full-transcriptome expression analysis for four wheat lines (Manitou and Norstar, winter and spring) during a long time. The authors noted that there is a group of genes that statistically significantly change their expression in response to the duration of cold stress and a different plant genotype. These genes are responsible for the transport, oxidation-reduction, and stress response processes. The study (Janmohammadi, Enayati & Sabaghnia, 2012) described that during the cold acclimatization, the winter wheat genotype CDC-Ospray was characterized by a significant increase in the activity of the enzymes SOD, APX, CAT, and guaiacol peroxidase, but not spring genotype Pishtaz. The authors argue that this may indicate a greater scavenging capacity of winter varieties. However, in a review article Caverzan, Casassola & Brammer (2016), the authors conclude that different types of stress affect the change in the number of AOS enzymes in different ways and H_2_O_2_ signaling is an important factor in the process of adaptation to stress.

Our qPCR analysis showed a genotype-specific reaction in stress conditions only for CAT B and CAT C for 24 h of cold treatment. Analysis of AOS genes for cv. S29 showed significant down-regulation of SOD B in 6 h treatment, upregulation of APX E, SOD E in 24 h treatment. For cv. YP down-regulation of SOD B in 6 h treatment was established and CAT C, APX D during 24 h of treatment; upregulation of CAT C (6 h) and CAT B, APX F (24 h). These results correspond to our meta-transcriptomic analysis for rice. In particular, the same genes SOD B and CAT C downregulated in rice stress-response clusters. However, different orthological groups were upregulated in the comparison between this data (APX E, APX F, SOD E, CAT B, CAT C) and meta-analysis of rice (APX H, GR A, GPX E, MDHAR A(OS09G39380)). Interestingly, that upregulated genes are presented by APXses but vary between other classes. So a meta-analysis with the involvement of a large number of replications allows a detailed description of the diversity in the transcriptional response for the genetic system and identify its stable components.

Note that some of AOS genes have a highly conservative character of evolution in cereals but do not reveal significant levels of expression in normal environmental conditions. Here we tested the hypothesis proposed in Doroshkov & Bobrovskikh (2018) about stress-induced upregulation for this type of genes. Namely, we experimentally detected a significant upregulation of a highly conservative APX (E and F) and SOD E genes in response to cold stress, whereas for rice SOD E is also significantly activated in response to water deficiency. Thus, these genes can be significant for plant adaptation to stressful conditions.

## Conclusions

It would seem that the AOS in response to an increased amount of ROS in stress conditions should increase the production of its enzymes. However, the complex assessment obtained by transcriptome meta-analysis and experiment study indicated the ambiguity in the regulation of the AOS components. Analysis of the transcriptome data for bread wheat showed that the exact measurements for antioxidant genes are incomplete, and we needed to set up our experiment to verify the obtained data. Comparison of orthologous groups of antioxidant genes according to rice and experimental data for wheat showed that in most cases orthologs are regulated differently depending on the types of stress exposure. However, some orthologs show a similar pattern of expression change (for example, SOD B shows significant downregulation in cold response at 6 h of experiment for bread wheat cultivars S29 and YP and in clusters of CR2, CR4 combining experiments for rice; DHAR B shows significant downregulation in experiment GSE23889 for bread wheat and in clusters CR1-CR5, combining experiments for rice). Thus, on the one hand, a transcriptome meta-analysis for rice showed that there are persistent patterns of stress response to cold and water deficiency, but on the other hand, the analyzed experiments from different researchers used different protocols for creating stress effects on plants, different conditions, and their cultivation, and also assumed the collection and analysis of various tissues, which certainly affects the resulting data. Generally, the cold response is characterized by compact clustering, comparable to the control cluster, whereas the response to water deficiency is mostly heterogeneous. Experimental analysis conducted on two genotypes of wheat more accurately describes the response of AOS genes on cold stress. The data are primarily similar between genotypes and time of exposure, which indicates a uniform pattern of regulation. We have shown that the approach that includes a comprehensive study using both available transcriptome data and experimentally obtained data, makes it possible to supplement and expand the features of such a complex genetic system, such as antioxidant, the dynamics of which is determined by both the genetic component and the environmental conditions.

## Supplemental Information

10.7717/peerj.7791/supp-1 Supplemental Information 1 The transcriptome experiments for rice (*Oryza sativa* L.) and (*Triticum aestivum* L.) from the GEO database used for meta-analysis. The “Organism” column contains the names of the species, the ID reflects the corresponding card number from the GEO database. The “Title” column contains the experiment titles given from the GEO accession pages. The “Conditions” column represents the details of the stress conditions used in the experiment. The “Type” column reflects the measurement technology for genes expression (microarray or next- generation sequencing). The “Tissue” column indicates a tissue used for expression analysis by researchers.

10.7717/peerj.7791/supp-2 Supplemental Information 2 The analysis of the antioxidant genes expression in rice. The data are given for the clusters of experimental replicates indicating cold response (CR) and water deficiency response (WDR) identified in the present work. The rows display the Fold Change of Gene expression values (red is for upregulated genes, green is for downregulated genes) and the corresponding confidence value counted for the gene (bold font is for reliable values). The Enzymatic class header breaks genes into relevant classes (ascorbate peroxidase (APX), catalase (CAT), dehydroascorbate reductase (DHAR), glutathione peroxidase (GPX), glutathione reductase (GR), monodehydroascorbate reductase (MDHAR), superoxide dismutase (SOD)).

10.7717/peerj.7791/supp-3 Supplemental Information 3 The analysis of the antioxidant genes expression in rice. The data are given for individual experiments. The Enzymatic class header breaks genes into relevant classes (ascorbate peroxidase (APX), catalase (CAT), dehydroascorbate reductase (DHAR), glutathione peroxidase (GPX), glutathione reductase (GR), monodehydroascorbate reductase (MDHAR), superoxide dismutase (SOD)).

10.7717/peerj.7791/supp-4 Supplemental Information 4 The relative expression ratio obtained by real-time PCR.

10.7717/peerj.7791/supp-5 Supplemental Information 5 Phylogenetic trees for seven classes of the AOS enzymes.

## Abbreviations

NGS: Next generation sequencing
AOS: Antioxidant system
ROS: Reactive oxygen species
ABA: Abscisic acid
SOD: Superoxide dismutase
CAT: Catalase
GPX: Glutathione peroxidase,
Asc-Gsh: Ascorbate-glutathione cycle
APX: Ascorbate peroxidase
DHAR: Dehydroascorbate reductase
MDHAR: Monodehydroascorbate reductase
GR: Glutathione reductase
AsA: Ascorbate
MDHA: Monodehydroascorbate
DHA: Dehydroascorbate
GSH: Glutathione
GSSG: Glutathione disulfide
ETC: Electron transport chain
CR: Cold response
WDR: Water deficiency response
S29: Saratovskaya
YP: Yanetzkis Probat
PCA: Principal component analysis
GOI: Genes of interest.

## Additional Information and Declarations

### Data Availability

The following information was supplied regarding data availability: The raw data was available in GEO: GSE21651, GSE23211, GSE24048, GSE25176, GSE26280, GSE37940, GSE38023, GSE41647, GSE4438, GSE54466, GSE64576, GSE65024, GSE65025, GSE67373, GSE6901, GSE71680, GSE74465, GSE78972, GSE80811, GSE81462, GSE83378, GSE83912, GSE23889, GSE30436, GSE31762, GSE42214, GSE45563, GSE47090, GSE79522. To perform meta-analysis we used a transcriptome data from the open GEO Datasets database, which were downloaded and processed by us in accordance with the protocol described in the materials and methods. The list of experiments is available in Table S1. Data with the following GEO accession numbers were used GSE21651, GSE23211, GSE24048, GSE25176, GSE26280, GSE37940, GSE38023, GSE41647, GSE4438, GSE54466, GSE64576, GSE65024, GSE65025, GSE67373, GSE6901, GSE71680, GSE74465, GSE78972, GSE80811, GSE81462, GSE83378, GSE83912, GSE23889, GSE30436, GSE31762, GSE42214, GSE45563, GSE47090, GSE79522.
