# Supplementary material for: Stress-induced changes in the expression of antioxidant system genes for rice (Oryza sativa L.) and bread wheat (Triticum aestivum L.)
Source: PeerJ. 2019 Nov 29;7:e7791. doi: 10.7717/peerj.7791 (PMC6886489; doi:10.7717/peerj.7791)
Supplement: Supplemental Information 4 [file peerj-07-7791-s004.docx]

**Table S4.** The relative expression ratio obtained by real-time PCR

| **variety** | **treat** | **CAT A** | **CAT B** | **CAT C** | **APX D** | **APX E** | **APX F** | **SOD A** | **SOD B** | **SOD E** |
| --- | --- | --- | --- | --- | --- | --- | --- | --- | --- | --- |
| **S29** | 6h cold | 0,8231 | 0,3775 | 1,8592 | 0,4954 | 1,5540 | 0,8918 | 0,4505 | 0,6105 | 1,2983 |
| **YP** |  | 0,9618 | 1,0148 | 2,1709 | 0,7629 | 2,0536 | 1,6777 | 0,6449 | 0,7872 | 1,3369 |
| **S29** | 24h cold | 0,8605 | 1,0570 | 0,5559 | 0,9847 | 2,9968 | 1,2647 | 1,5201 | 1,1604 | 2,0178 |
| **YP** |  | 0,8012 | 5,0597 | 0,0064 | 0,1119 | 1,3666 | 2,1592 | 2,6151 | 1,0435 | 1,5572 |
