# Supplementary material for: Stress-induced changes in the expression of antioxidant system genes for rice (Oryza sativa L.) and bread wheat (Triticum aestivum L.)
Source: PeerJ. 2019 Nov 29;7:e7791. doi: 10.7717/peerj.7791 (PMC6886489; doi:10.7717/peerj.7791)
Supplement: Supplemental Information 5 [file peerj-07-7791-s005.pptx]

## Slide 1
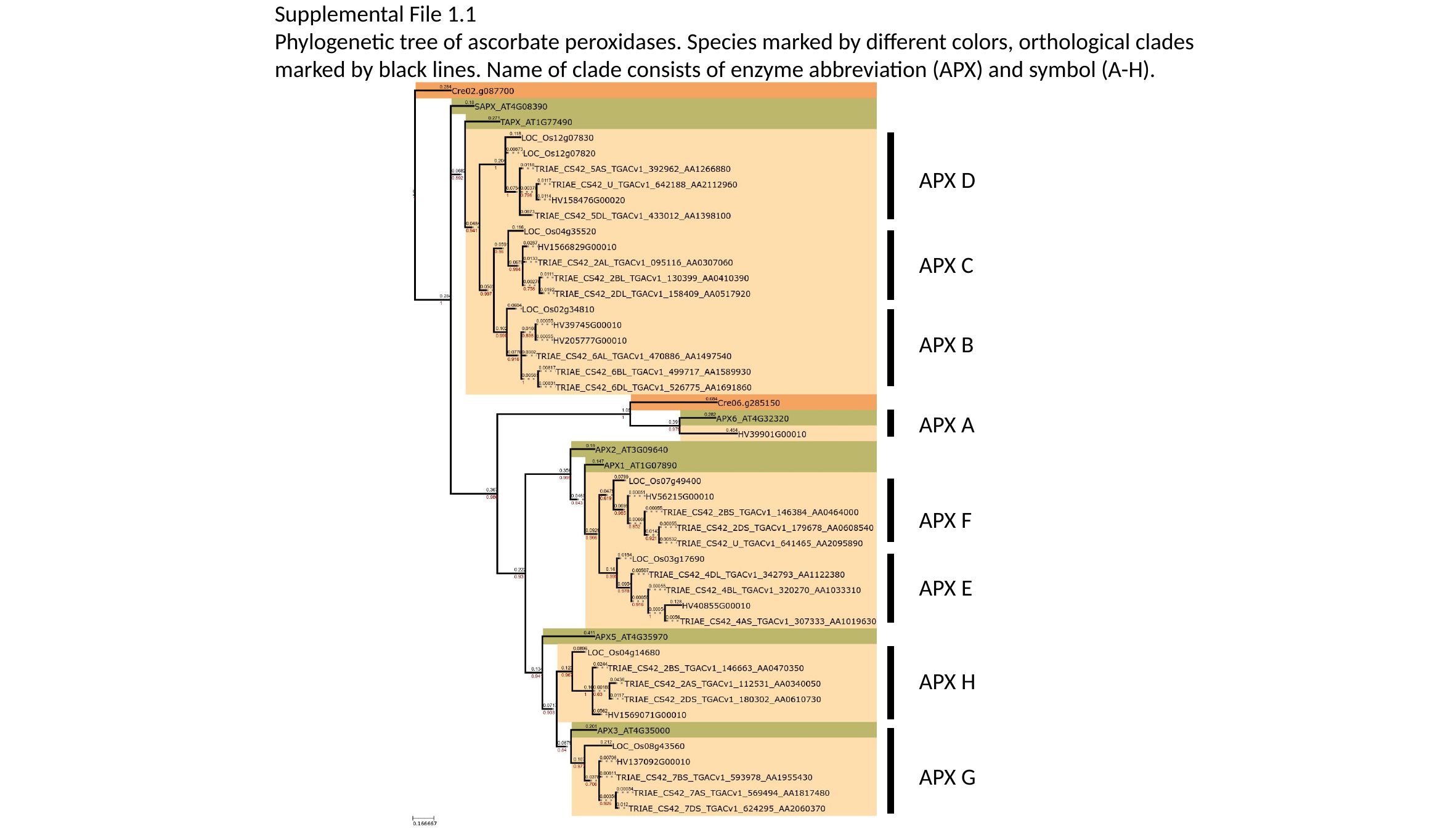

Supplemental File 1.1
Phylogenetic tree of ascorbate peroxidases. Species marked by different colors, orthological clades marked by black lines. Name of clade consists of enzyme abbreviation (APX) and symbol (A-H).
APX D
APX C
APX B
APX A
APX F
APX E
APX H
APX G

## Slide 2
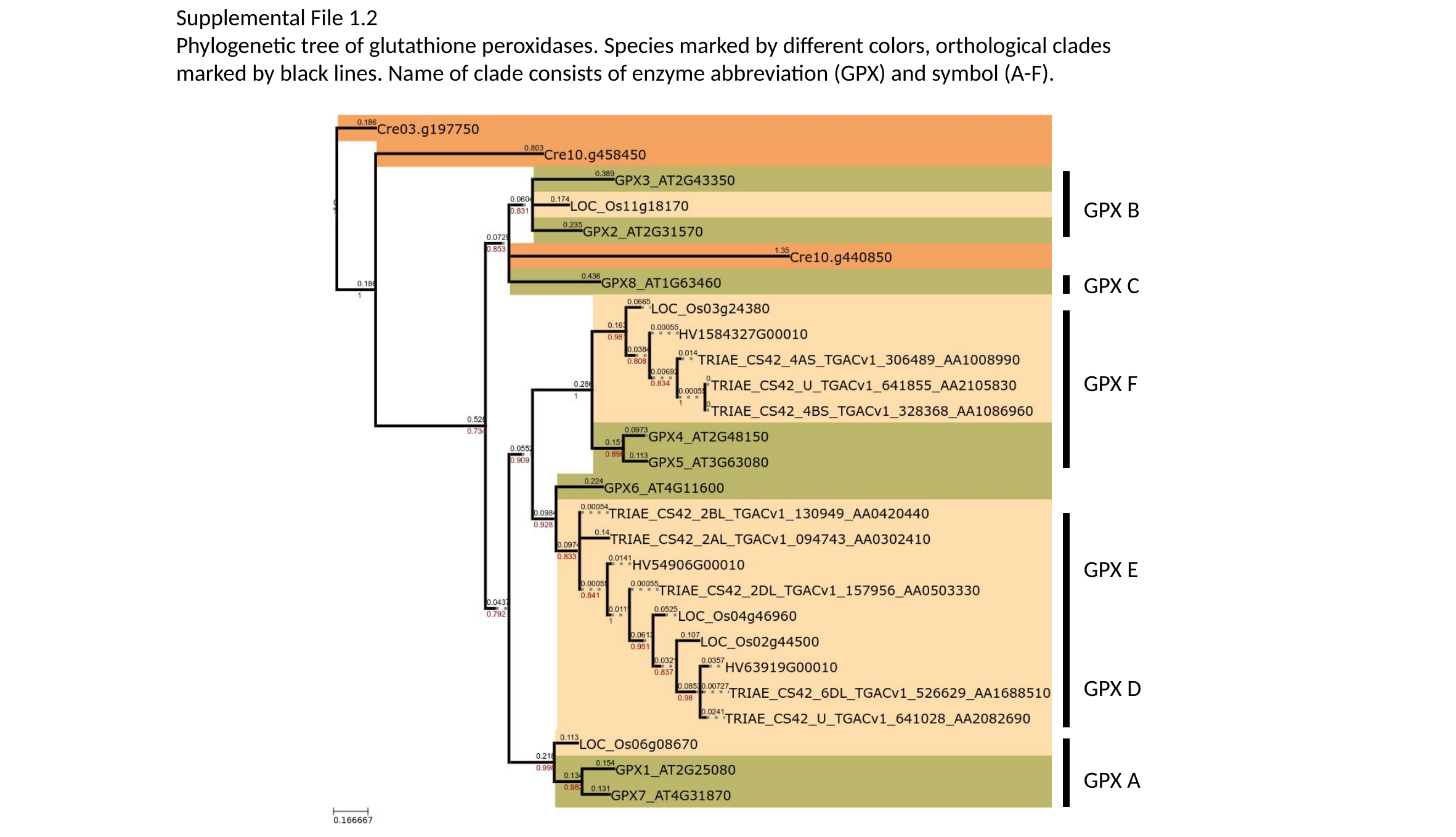

Supplemental File 1.2
Phylogenetic tree of glutathione peroxidases. Species marked by different colors, orthological clades marked by black lines. Name of clade consists of enzyme abbreviation (GPX) and symbol (A-F).
GPX B
GPX C
GPX F
GPX E
GPX D
GPX A

## Slide 3
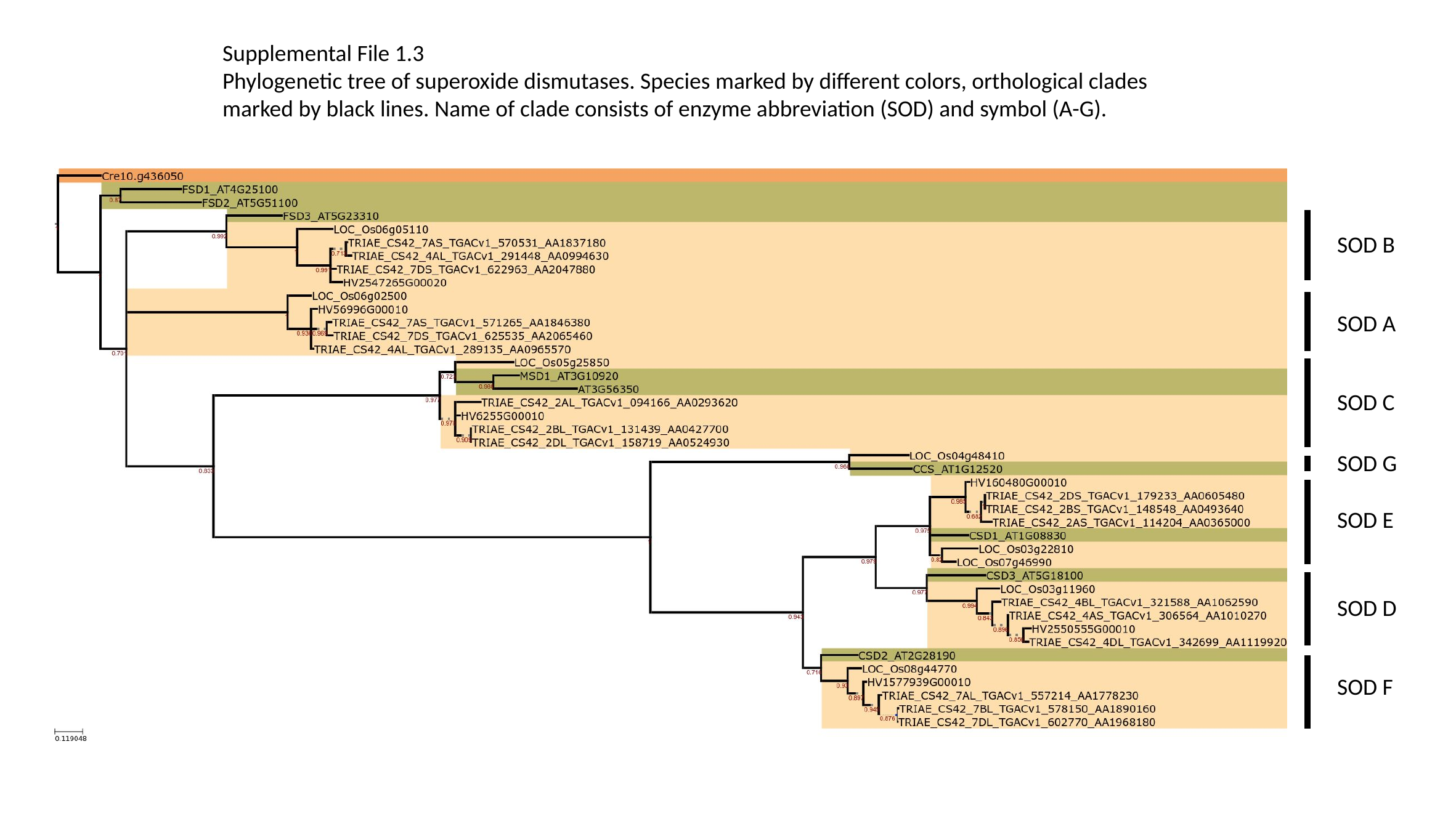

Supplemental File 1.3
Phylogenetic tree of superoxide dismutases. Species marked by different colors, orthological clades marked by black lines. Name of clade consists of enzyme abbreviation (SOD) and symbol (A-G).
SOD B
SOD A
SOD C
SOD G
SOD E
SOD D
SOD F
CAT B
CAT A
CAT C

## Slide 4
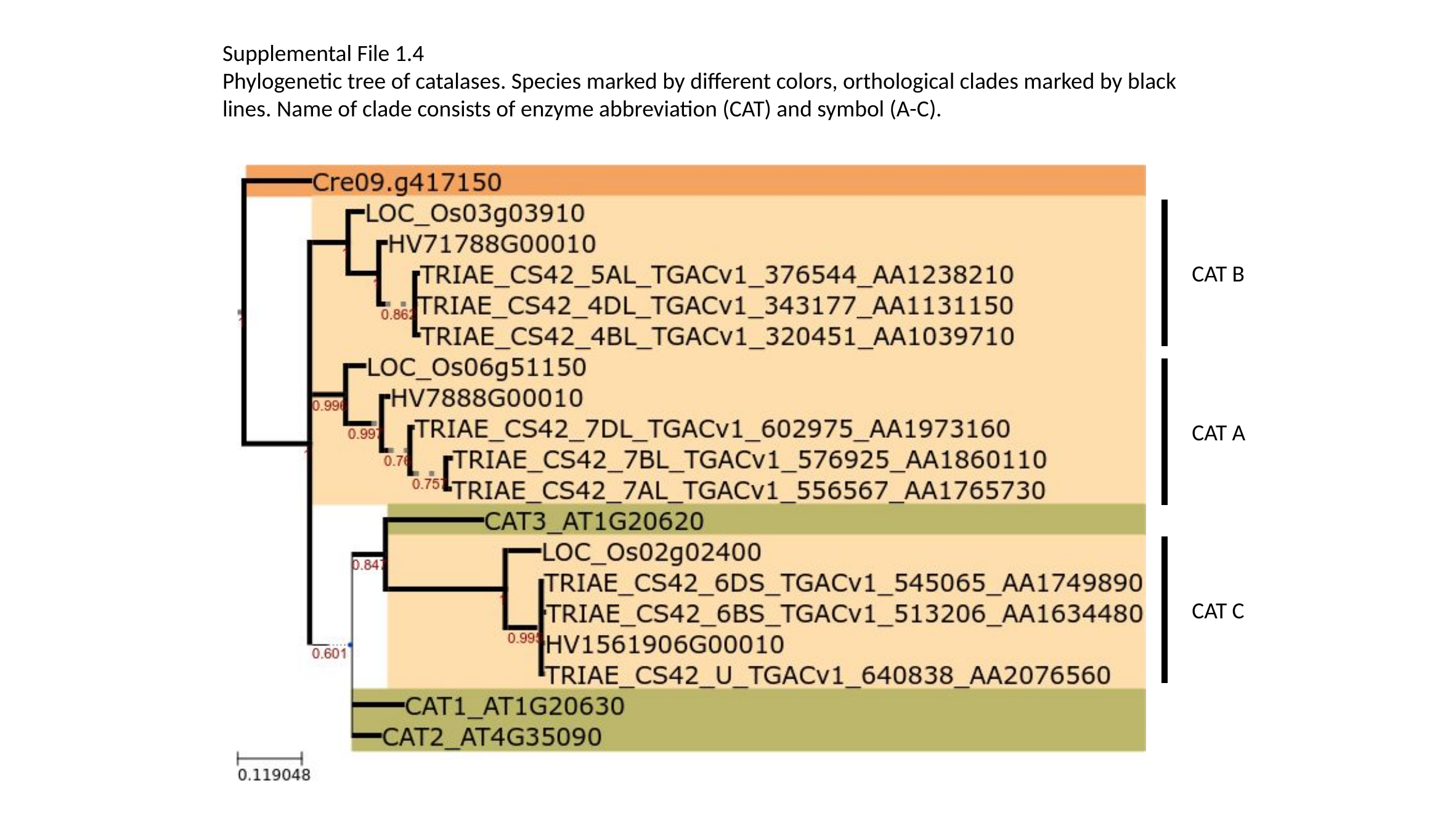

Supplemental File 1.4
Phylogenetic tree of catalases. Species marked by different colors, orthological clades marked by black lines. Name of clade consists of enzyme abbreviation (CAT) and symbol (A-C).
CAT B
CAT A
CAT C

## Slide 5
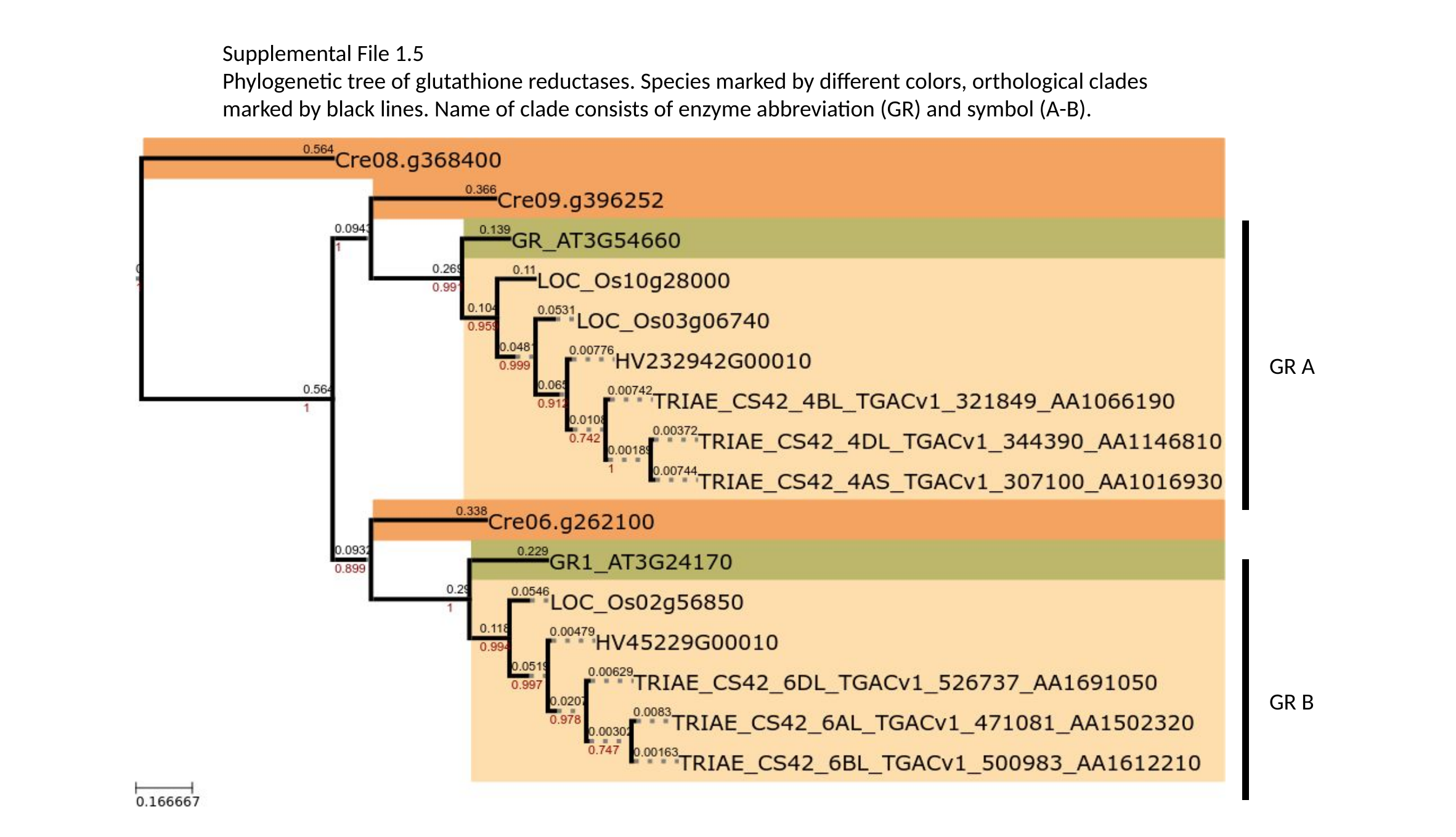

Supplemental File 1.5
Phylogenetic tree of glutathione reductases. Species marked by different colors, orthological clades marked by black lines. Name of clade consists of enzyme abbreviation (GR) and symbol (A-B).
GR A
GR B

## Slide 6
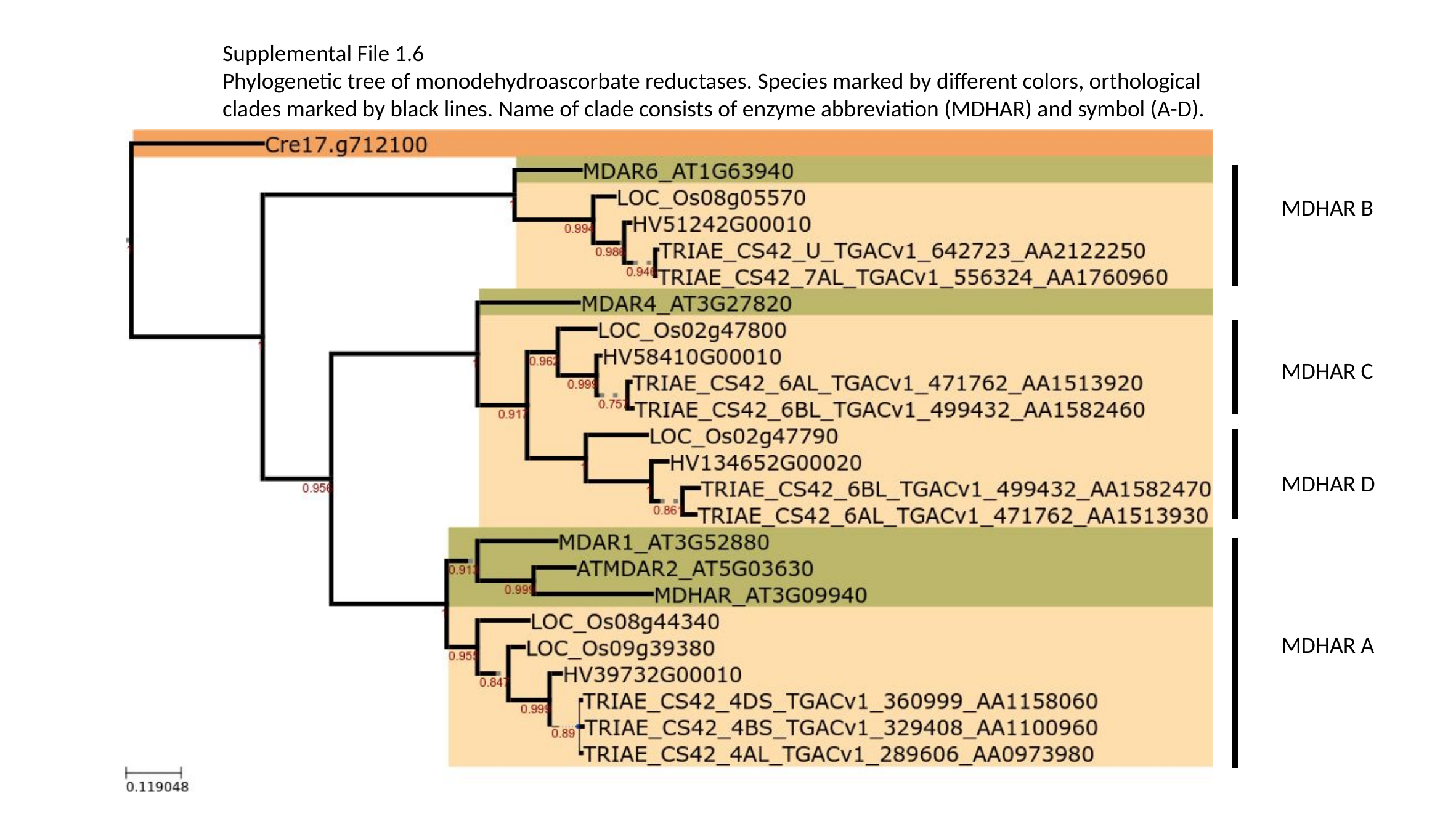

Supplemental File 1.6
Phylogenetic tree of monodehydroascorbate reductases. Species marked by different colors, orthological clades marked by black lines. Name of clade consists of enzyme abbreviation (MDHAR) and symbol (A-D).
MDHAR B
MDHAR C
MDHAR D
MDHAR A

## Slide 7
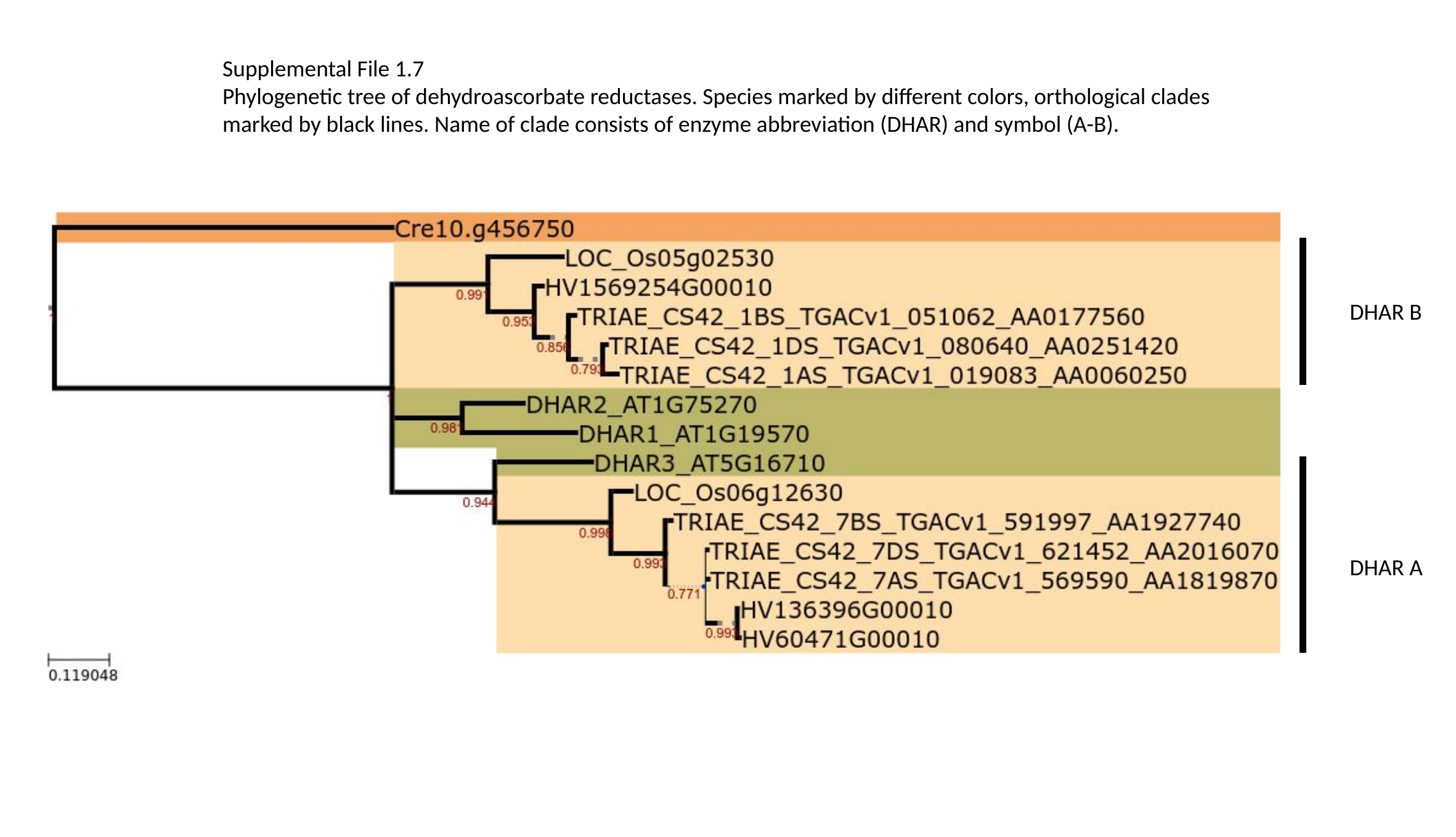

Supplemental File 1.7
Phylogenetic tree of dehydroascorbate reductases. Species marked by different colors, orthological clades marked by black lines. Name of clade consists of enzyme abbreviation (DHAR) and symbol (A-B).
DHAR B
DHAR A
